# Does Dysphagia Indicate Recurrence of Benign Esophageal Strictures?

**DOI:** 10.1155/DTE.2.7

**Published:** 1995

**Authors:** Olle Ekberg, Anders Borgström, Frans-Thomas Fork, Eje Lövdahl

**Affiliations:** 1 Department of Diagnostic Radiology Malmö General Hospital University of Lund Malmö S-214 01 Sweden; 2 Department of Surgery Malmö General Hospital University of Lund Malmö S-214 01 Sweden; 3 Endoscopy unit Malmö General Hospital University of Lund Malmö S-214 01 Sweden

## Abstract

Esophageal dilatation in dysphagic patients with benign strictures is usually considered successful if the patients' dysphagia is alleviated. However, the relation between dysphagia and the diameter of a stricture is not well understood. Moreover, the dysphagia may also be caused by an underlying esophageal motor disorder. In order to compare symptoms and objective measurements of esophageal stricture, 28 patients were studied with interview and a radiologic esophagram. The latter included swallowing of a solid bolus. All patients underwent successful balloon dilatation at least one month prior to this study. Recurrence of a stricture with a diameter of less than 13 mm was diagnosed by the barium swallow in 21 patients. Recurrence of dysphagia was seen in 15 patients. Thirteen patients denied any swallowing symptoms. Chest pain was present in 9 patients. Of 15 patients with dysphagia 2 (13%) had no narrowing but severe esophageal dysmotility. Of 13 patients without dysphagia 9 (69%) had a stricture with a diameter of 13 mm or less. Of 21 patients with a stricture of 13 mm or less 14 (67%) were symptomatic while 7 (33%) were asymptomatic. Four of 11 patients with retrosternal pain had a stricture of less than 10 mm. Three patients with retrosternal pain and obstruction had severe esophageal dysmotility. Whether or not the patients have dysphagia may be more related to diet and eating habits than to the true diameter of their esophageal narrowing. We conclude that the clinical history is non-reliable for evaluating the results of esophageal stricture dilatation. In order to get an objective measurement of therapeutic outcome, barium swallow including a solid bolus is recommended.


*Diagnostic and Therapeutic Endoscopy*, 1995, Vol. 2, pp. 7–9
Reprints available directly from the publisher
Photocopying permitted by license only

© 1995 Harwood Academic Publishers GmbH
Printed in Singapore

### Does Dysphagia Indicate Recurrence of Benign Esophageal Strictures?

OLLE EKBERG,[1] ANDERS BORGSTRÖM,[2] FRANS-THOMAS FORK[1,3] and EJE LÖVDAHL[1,3]

*From the Department of Diagnostic Radiology,[1] the Department of Surgery,[2] and the Endoscopy unit,[3] Malmö General Hospital, University of Lund, S-214 01 Malmö, Sweden*

*(Received December 19, 1994; in final form March 3, 1995)*

Esophageal dilatation in dysphagic patients with benign strictures is usually considered successful if the patients' dysphagia is alleviated. However, the relation between dysphagia and the diameter of a stricture is not well understood. Moreover, the dysphagia may also be caused by an underlying esophageal motor disorder. In order to compare symptoms and objective measurements of esophageal stricture, 28 patients were studied with interview and a radiologic esophagram. The latter included swallowing of a solid bolus. All patients underwent successful balloon dilatation at least one month prior to this study. Recurrence of a stricture with a diameter of less than 13 mm was diagnosed by the barium swallow in 21 patients. Recurrence of dysphagia was seen in 15 patients. Thirteen patients denied any swallowing symptoms. Chest pain was present in 9 patients. Of 15 patients with dysphagia 2 (13%) had no narrowing but severe esophageal dysmotility. Of 13 patients without dysphagia 9 (69%) had a stricture with a diameter of 13 mm or less. Of 21 patients with a stricture of 13 mm or less 14 (67%) were symptomatic while 7 (33%) were asymptomatic. Four of 11 patients with retrosternal pain had a stricture of less than 10 mm. Three patients with retrosternal pain and obstruction had severe esophageal dysmotility. Whether or not the patients have dysphagia may be more related to diet and eating habits than to the true diameter of their esophageal narrowing. We conclude that the clinical history is non-reliable for evaluating the results of esophageal stricture dilatation. In order to get an objective measurement of therapeutic outcome, barium swallow including a solid bolus is recommended.

**KEY WORDS:** Esophagus, esophagitis, reflux, function, interventional procedure, motility, stenosis or obstruction

### INTRODUCTION

Benign strictures of the esophagus can be treated with balloon dilatation under endoscopic and/or fluoroscopic control. Immediate relief of obstructive symptoms is reported in 70–90%, while 60% of the patients are asymptomatic three months after the procedure.[1–7] The patients' symptoms may not readily correlate with the diameter of the stricture. Dysphagia including a feeling of obstruction may be due to esophageal dysmotility which might coincide with any esophageal stricture, especially in patients with gastroesophageal reflux disease. Any evaluation of stricture dimensions is difficult during endoscopy. We, therefore, hypothesized that a careful radiologic examination could be used for triage of these patients. To that effect we adopted a dedicated radiologic technique including swallowing of a solid tablet for assessment of esophageal strictures.

The aim of the present study was therefore to correlate symptoms of dysphagia and dietary restrictions to the "true" diameter of the esophageal stricture and esophageal dysmotility in patients with benign esophageal strictures, earlier treated by balloon dilatation.

### MATERIALS AND METHODS

Forty-three patients underwent balloon dilatation of benign strictures of the esophagus during a 4-year period. They were all invited to a follow-up examination. Twenty-eight patients complied to a complete follow-up performed 0.5 to 3 years after the dilatation. These did not

Address for correspondence: Olle Ekberg, MD, Department of Diagnostic Radiology, University of Lund, Malmö General Hospital, S-214 01 MALMö, Sweden.

differ in any respect from those who did not comply. All had become asymptomatic immediately post-dilatation. The dilatation had been performed at least one month earlier. Twenty-four patients had strictures due to gastroesophageal reflux disease, 2 due to sclerotherapy of varices. One had a stricture due to longstanding nasogastric intubation, and one had a stricture of a postoperative anastomosis between the esophagus and jejunum.

The follow-up included an interview according to a standardized protocol where the symptoms were scored on a six-grade scale according to Earlam and Cunha-Melo.[8] The radiologic examination of the esophagus was performed as a biphasic study. The esophageal anatomy was documented by a double contrast examination with a high density (240% w/v) barium suspension and the esophageal function was evaluated using a low density (60% w/v) barium suspension with the patient recumbent. Assessment of esophageal function was done according to well-known criteria.[9] All patients were also given a solid round tablet (diameter 13 mm) for detection of narrowings and evaluation of propulsion.[10] The geometric enlargement was determined by the same solid tablet and the size of any narrowing therefore was corrected. The patient was also instructed to report any symptoms of obstruction during the tablet swallow. The follow-up period ranged from 1 to 57 months, median 14. Median age among screenees was 72 years, range 31–90 years.

Statistical analysis was done using the CHI 2-test.

### RESULTS

After dilatation fifteen patients experienced obstruction during eating from time to time, while 13 denied any swallowing symptoms. Chest-pain was present in 9 patients.

The correct width of the lumen of radiographically demonstrated stenosis is graphically demonstrated in Figure 1. In 21 patients a stricture less than 13 mm in width was diagnosed.

Of 15 patients with dysphagia two (13%), had no narrowing but severe esophageal dysmotility.

Of 13 patients without dysphagia 9 (69%) had a stricture with a diameter of 13 mm or less.

Of 21 patients with a stricture of 13 mm or less, 14 (67%) were symptomatic while 7 (33%) were asymptomatic. Of 16 patients with a narrowing of 9 mm or less 6 (38%) were asymptomatic.

Only 4 of 9 patients with retrosternal pain had a stricture of less than 10 mm. Three patients with retrosternal pain and obstruction had the most severe esophageal dysmotility.

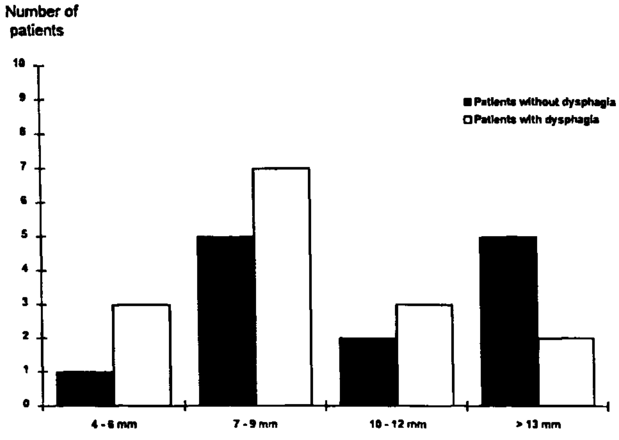

**Figure 1** Diameter of the esophageal narrowing.

In 21 of the 28 patients the tablet got stuck. This caused symptoms of obstruction in only 3. In 2 of the 3 patients there was a stricture while 1 had esophageal dysmotility.

None of the differences reached a statistical significance of $p < 0.05$.

### DISCUSSION

The importance of the clinical history in diagnosing and revealing the etiology of esophageal symptoms has been pointed out. By a careful interview and using a custom-tailed algorithm for the assessment of dysphagia a correct diagnosis can be revealed in 85% of patients with dysphagia.[11] This was an even higher percentage than radiology would match in the same study. Patients with dysphagia may seek medical advice long before the correct diagnosis has been revealed. This may, of course, be due to reluctance from the clinician in taking a correct clinical history or ordering the correct examination for confirmation of a suspected underlying disease. However, other patients became frustrated over time not having gained attention to their complaints. In a recent study, Gustavsson showed that dysphagia patients tended to denial and/or concealment of dysphagia.[12] However, there is still another category of dysphagic patients who adjust or compensate for an abnormality in the swallowing apparatus. This has been clarified by Buchholz who showed that this may lead to a complete loss of symptoms despite severe loss of function.[13] The aim of the present study was to analyze the symptoms in patients treated with dilatation of benign esophageal strictures, and to compare symptoms with the dimension of the stricture.

There has been a tendency to use the patients' symptoms as a standard for assessing the results of balloon dilatation of benign esophageal strictures. However, in order to obtain an objective measurement of the width after treatment of the stricture, radiology of the esophagus was used.

The periods between esophageal dilatation and follow-up varied but did not influence the aim of the study, namely to compare dysphagia of a benign esophageal stricture with symptoms of obstruction during swallowing.

The normal esophagus when well distended has a width of 2–3 cm.[14] A stricture diameter of 13 mm or less has often been regarded as crucial.[14,15] Our results show that even a tight esophageal stricture (≤ 13 mm) is frequently (38%) asymptomatic and therefore the absence of dysphagia is not a sensitive indicator of successful dilatation. Not even in those with a diameter of 6 mm or less was dysphagia always present. This was surprising since the minimal diameter tolerated is stated to be not less than 13 mm.[8,16]

There might be at least two explanations for this observation. Firstly, we are routinely recording measurements corrected for the radiographic magnification, i.e., our figures might not be comparable with those in the literature; secondly, patients may get accustomed to their problems and adjust their swallowing and eating habits; and thirdly, patients may deny symptoms in order to avoid the unpleasantness of yet another dilatation procedure. In our experience most patients do not spontaneously include change in diet and eating habits under "dysphagia—symptoms", which to them merely seems to infer "feeling-of-obstruction-when-eating".

However, it may very well be that even a minimal increase in width of the stricture is enough to make the patients asymptomatic as long as they stick to their restricted diets and eating habits. It is, therefore, necessary to use objective criteria like barium swallow to evaluate the short- and long-term results of dilatation. Treatment should basically be guided by the patients symptoms. However, a patient with a tight stricture although asymptomatic, has an increased risk of acute obstruction by bolus impaction. Therefore, monitoring long-term results for comparison of different treatments and regimins ought to include objective measurement as a complement to the clinical history. On the other hand patients with a wide esophagus might have severe dysphagia similar to those with strictures. In these patients esophageal dysmotility is commonly the cause of dysphagia.

The additional use of the tablet as part of our routine protocol has only slightly increased the examination time. In fact our impression has been that one rather immediately reaches to a correct diagnosis and therefore refrains from protracted fluoroscopy during multiple liquid barium swallows. Many patients were, however, at first reluctant to swallowing the tablet which they claimed would stick. It had to be explained to them that this was the objective of the test. The tablet is readily available with an antacid effect and harmless to the mucosa.

**Table 1** Number of patients with symptoms, score 1–6, at follow-up interview

| *Score* | *Symptoms* | *At follow-up interview* |
|---|---|---|
| 1 | No symptom | 13 |
| 2 | Difficulty swallowing solids | 11 |
| 4 | Difficulty swallowing minced or pureed food | 3 |
| 6 | Difficulty swallowing liquids | 1 |
| Total number of patients. | | 28 |

Therefore, any follow-up of balloon dilatation of benign esophageal strictures ought to include an objective measurement. Barium swallow with the additional use of an inert tablet with a known diameter offers a simple modality to achieve such an objective measurement.

### REFERENCES

1. Wesdorp ICE, Huibregtse K, Tytgat GN. Results of conservative treatment of benign esophageal strictures. A follow-up study in 100 patients. Gastroenterology, 1982;82:487.
2. Bradpiece HA, Galland RB, Spencer J. Esophageal dilatation as an outpatient procedure. Surg Gynec Obstet, 1988;167:45–48.
3. Dumon JR, Meric B, Sivak MV, Fleischer D. A new method of esophageal dilatation using Savary-Gilliard bougies Gastrointest Endosc, 1985;31:379–382.
4. Graham DY, Tabibian N, Schwartz JT, Smith JL. Evaluation of the effectiveness of through-the-scope balloons as dilators of benign and malignant gastrointestinal strictures. Gastrointest Endosc, 1987;33:432–435.
5. Ogilvie AC, Ferguson R, Atkinson M. Outlook with conservative treatment of oesophageal stricture. Gut, 1980;21:23–25.
6. Patterson DJ et al. Natural history of benign esophageal stricture treated by dilatation. Gastroenterology, 1983;85:346–350.
7. Kozarek RA. Hydrostatic balloon dilation of gastrointestinal stenoses: a national survey. Gastrointest Endoscopy, 1986;32:15–19.
8. Earlam R, Cunha-Melo JR. Benign oesophageal strictures: historical and technical aspects of dilatation. Br J Surg, 1981;68:829–836.
9. Gelfand DW. Radiologic evaluation of the pharynx and esophagus. In: Dysphagia Diagnosis and treatment. Ed Gelfand DW and Richter JE Igaku-Shoin New York 1989 p 31–81.
10. van Westen D, Ekberg O. Solid bolus swallowing in the radiologic evaluation of dysphagia. Acta Radiol, 1993;34:372–375.
11. Edwards DAW. Discrimatory value of symptoms in the differential diagnosis of dysphagia. Clinics in Gastroenterology, 1976;5:49–57.
12. Gustavsson B, Tibbling L. Dysphagia, an unrecognized handicap. Dysphagia, 1991;6:193–199.
13. Buchholz DW, Bosma JF, Donner MW. Adaptation, compensation, and decompensation of pharyngeal swallow. Gastrointest Radiol, 1985;10:235–239.
14. Stewart ET, Dodds WJ. Radiology of the esophagus. In Margulis and Burhenne's: Alimentary Tract Radiology. Ed. Freeny PC, Stevenson GW. Mosby, 1994;192–263.
15. Schatzki R. The lower esophageal ring: Long term follow-up of symptomatic and asymptomatic rings. AJR, 1963;90:805–815.
16. Tytgat GNJ. Dilation therapy of benign esophageal stenoses. World J Surg, 1989;13:142–148.

